# Socio-demographic and behavioural correlates of physical activity perception in individuals with recently diagnosed diabetes: results from a cross-sectional study

**DOI:** 10.1186/1471-2458-13-678

**Published:** 2013-07-24

**Authors:** Gráinne H Long, Søren Brage, Nicholas J Wareham, Esther MF van Sluijs, Stephen Sutton, Simon J Griffin, Rebecca K Simmons

**Affiliations:** 1 MRC Epidemiology Unit, University of Cambridge, Institute of Public Health, Cambridge, UK; 2 UKCRC Centre for Diet and Activity Research, Institute of Public Health, Cambridge, UK; 3 Behavioural Science, Institute of Public Health, University of Cambridge, Cambridge, UK

**Keywords:** Type 2 diabetes mellitus, Physical activity, Overestimation, Awareness, Perception, Health behaviour, Lifestyle behaviour, Social correlates of health, Health promotion intervention

## Abstract

**Background:**

Physical activity (PA) levels in type 2 diabetes mellitus (T2DM) patients are generally low. Poor PA perception may impede healthy behaviour change in this high risk group. We describe (*i*) objective PA levels, (*ii*) the difference between objective and self-reported PA (‘PA disparity’) and the correlates of (*iii*) PA disparity and (*iv*) overestimation in recently diagnosed T2DM patients.

**Methods:**

Cross-sectional analysis of 425 recently diagnosed T2DM patients aged 42 to 71, participating in the *ADDITION-Plus* study in Eastern England, UK. We define ‘PA disparity’ as the non-negative value of the difference (in mathematical terms the absolute difference) between objective and self-reported physical activity energy expenditure (PAEE in kJ · kg^-1^ · day^-1^). ‘Overestimators’ comprised those whose self-reported- exceeded objective-PAEE by 4.91 kJ · kg^-1^ · day^-1^(the equivalent of 30 minutes moderate activity per day). Multivariable linear regression examined the association between PA disparity (continuous) and socio-demographic, clinical, health behaviour, quality of life and psychological characteristics. Logistic regression examined the association between PA overestimation and individual characteristics.

**Results:**

Mean objective and self-reported PAEE levels ± SD were 34.4 ± 17.0 and 22.6 ± 19.4 kJ · kg^-1^ · day^-1^, respectively (difference in means =11.8; 95% CI = 9.7 to 13.9 kJ · kg^-1^ · day^-1^). Higher PA disparity was associated with male sex, younger age, lower socio-economic status and lower BMI. PA overestimators comprised 19% (n = 80), with those in routine/manual occupations more likely to be overestimators than those in managerial/professional occupations.

**Conclusions:**

T2DM patients with poor physical activity perception are more likely to be male, younger, from a lower socio-economic class and to have a lower BMI. PA overestimators were more likely to be in lower socio-economic categories. Self-monitoring and targeted feedback, particularly to those in lower socio-economic categories, may improve PA perceptions and optimise interventions in T2DM patients. Our findings suggest that strategies for enabling realistic assessment of physical activity levels, through self-monitoring or feedback, warrant further investigation and may help refine and improve physical activity interventions.

## Background

Individuals with type 2 diabetes mellitus (T2DM) are routinely advised about the importance of physical activity (PA) for controlling risk factors associated with disease progression [1,2]. PA positively affects glycaemic control in individuals with established T2DM [3-5] and as part of multifactorial interventions, is associated with significant improvements in blood pressure, cholesterol, weight loss and fitness [6]. Less is known regarding the effectiveness of PA interventions adopted early in the disease trajectory, for example following screen-detection [7]. Recently diagnosed T2DM patients represent a group of individuals with high, but modifiable cardiovascular risk [8]. It is plausible that building or reinforcing good PA habits early in the disease trajectory may help prevent or delay complications associated with T2DM.

Evidence suggests that recently diagnosed T2DM patients and those at high risk of T2DM have low PA levels, but data is limited [7,9,10]. In addition, poor recognition of PA inactivity may act as a barrier to healthy behaviour change in these individuals. For example, individuals who overestimate their PA activity are less likely to perceive a need to change their PA behaviour and may be unreceptive to health promotion interventions. Previous studies assessing PA awareness – in terms of the difference between an individual’s belief of meeting PA guidelines versus measured PA levels – report that between 15% and 35% of healthy adults are PA overestimators [11-13]. One study of individuals at high risk of T2DM objectively classified 63% as inactive, with individuals more likely to be PA overestimators if they were male, had low BMI and were from a low socio-economic background [14]. All previous studies categorise PA awareness and therefore do not fully capture the difference between true- and self reported- PA. Furthermore, most previous studies compared self-reported and self-rated measures of PA behaviour, which could lead to a biased assessment of agreement due to correlated error introduced by using measurement tools of the same fundamental type; an issue explored extensively in nutritional epidemiology [15,16].

Here we aimed to characterise objective PA levels using a combined heart rate and accelerometry monitor, assess the difference between continuously measured objective and self-reported PA and investigate associated characteristics in recently diagnosed T2DM from the *ADDITION-Plus* cohort [17]. We define PA disparity as the non-negative value of the difference (in mathematical terms the absolute difference) between objective and self-reported PAEE. Specifically, we *(i)* describe objectively measured free-living PA; (*ii*) describe PA disparity; (*iii*) examine which individual characteristics – from a range of socio-demographic, clinical, health behaviour, quality of life and psychological – are associated with PA disparity; and due to their potential public health importance, (*iv*) examine which characteristics are associated with PA overestimation. This will further our understanding of PA perceptions and may help optimize and target interventions promoting PA in recently diagnosed T2DM patients.

## Methods

### Study design and population

Full details of the study have been reported [17]. Briefly, *ADDITION-Plus* is a randomised controlled trial nested within the intensive treatment arm of the *ADDITION-Cambridge* study, which evaluated the efficacy of a facilitator-led, theory-based behaviour change intervention for recently diagnosed T2DM patients. Thirty four general practices in East Anglia participated. Eligible individuals were those aged 40 to 69 diagnosed with T2DM following screening or clinically diagnosed in the past 3 years in participating general practices. All participants received advice regarding the importance of a healthy lifestyle, including PA, for the control of diabetes, as well as a target for behaviour change (for PA, a gradual increase to reach the equivalent of 35 minutes of brisk walking per day, 7 days per week). Data were collected between 2003 and 2007 and analyzed in 2012. Exclusion criteria included women who were pregnant/lactating, those suffering from a psychotic illness or those with a prognosis of less than one year. Out of 1109 eligible participants, 478 agreed to participate in *ADDITION-Plus* and were individually randomised to receive either intensive treatment alone (n = 239), or intensive treatment plus the facilitator-led individual behaviour change intervention (n = 239) [17]. One year follow-up data was used in these analyses and health assessments at one year included physiological and clinical measurements, venesection by trained staff following standard operating procedures and the self-completion of questionnaires. Ethical approval was obtained from the Eastern Multi-Centre Research Ethics Committee (reference number: 02/5/54). Participants gave written informed consent. The trial is registered as ISRCTN 99175498.

### Outcome measurement

Both our objective and self-reported physical activity assessments capture total habitual physical activity. Free-living PA was objectively measured using a combined heart rate (HR) and accelerometry (ACC) monitor (Actiheart, CamNtech, Papworth, UK), worn continuously for 4 days [18]. This objective measure of physical activity was only available at one year of follow-up. Where possible, individual HR calibration was completed using a 15-minute graded-treadmill walk calibration [19]. A robust Gaussian process model inferred the latent HR time-series [20] and activity intensity (J · min^-1^ · kg^-1^) was estimated using a branched equation framework [21]. Resulting time-series data were summarised into mean physical activity energy expenditure over 24-hours (PAEE in kJ · kg^-1^ ·day^-1^). Where individual calibration measures were unavailable (n = 61), calibration of HR was derived across all valid study calibration tests using age, sex, beta blockage, and sleeping HR as pseudo-individual calibration.

**Figure 1:**
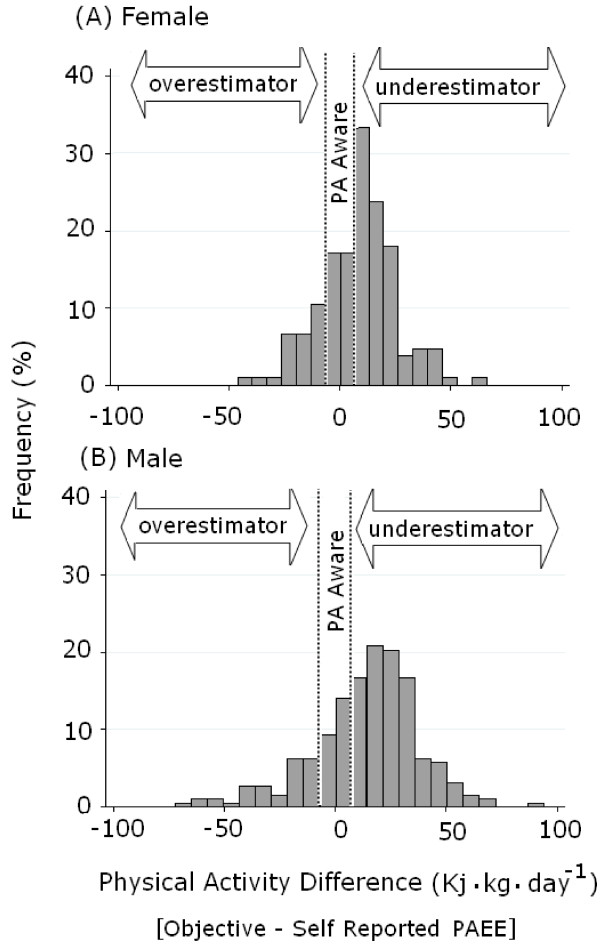
**Distribution of physical activity difference.** The difference between objective minus self-reported physical activity energy expenditure (PA difference) in recently diagnosed **(A)** female and **(B)** male T2DM patients is presented. Data were collected in Eastern England, UK between 2003 and 2007 (*ADDITION-Plus* study).

The previously validated short European Prospective Investigation into Cancer and Nutrition [EPIC]-Norfolk Physical Activity Questionnaire [EPAQ2] was used to measure self-reported past-year PA across four different domains (recreational, home activities, commuting/travel and occupational PA) [22]. Net Activity MET hrs · day^-1^ was computed by multiplying activity duration by the metabolic cost of each activity (activity-specific MET) [23,24], while discounting the resting metabolic rate. Daily PAEE estimates were derived from self-reported Net Activity MET hrs · day^-1^ using a method similar to that previously published [25,26], using [(1440 min·day^-1^) x (reported non-sedentary time/reported awake time)] as the scaling factor for average intensity. The difference between objective and self-reported PAEE (PA difference) was calculated for each individual (and can have positive or negative values, Figure 1A & B). The PAEE equivalent to 30 minutes of moderate activity was calculated as 4.91 kJ · kg^-1^· day^-1^ (MET equivalent of 3.3 METs for moderate walking at 3 miles · hr^-1^[23]) and was used to define a ‘PA Aware’ zone that included PA difference values falling within 4.91 kJ · kg^-1^· day^-1^ of zero (Figure 1A & B). Thus ‘overestimators’ comprised those whose self-reported exceeded their objective PAEE by 4.91 kJ · kg^-1^· day^-1^ and ‘underestimators’ comprised those whose objective exceeded their self-reported PAEE by 4.91 kJ · kg^-1^· day^-1^. ‘PA disparity’ was defined as the absolute difference between objective and self-reported PAEE. Individuals with missing self-reported or objective PA data (n = 51) and extreme PA disparity outliers (values >4 times the SD: n = 2), were not included in these analyses.

### Explanatory variables and covariates

Body height and weight were measured in individuals in light clothing, without shoes, using a fixed rigid stadiometer and scale (SECA, UK). A BMI greater than or equal to 25 or 30 kg/m^2^ defined overweight and obese, respectively [27]. Waist circumference was estimated as the average of two measurements taken with a tape measure halfway between the rib cage lowest point and the anterior superior iliac crests. HbA_1c_ was analysed in venous samples by ion-exchange high-performance liquid chromatography (Tosoh Bioscience, Redditch, UK). Total cholesterol was measured using standard enzymatic techniques [17]. Blood pressure was calculated as the mean of three automatic sphygmomanometer (Omron M4, UK) measurements performed after at least 10 minutes rest with participants seated and the cuff at heart level on the dominant arm.

Standardised questionnaires collected information on socio-demographic characteristics and lifestyle habits (smoking status, alcohol consumption). Socio-economic categories were based on the National Statistics Socio-Economic Classification (NS-SEC) and comprised: *(i)* managerial/professional; *(ii)* intermediate or *(iii)* routine/manual occupations based on current or previous occupation. A validated FFQ was used to estimate daily dietary intake [28]. The EuroQol (EQ-5D) measured health utility and health-related quality of life (QoL) [29,30]. The Audit of Diabetes-Dependent QoL (ADDQoL) measured respondents’ perceptions of the impact of T2DM on their QoL [31]. The generic Short Form 36-item (SF-36) Health Survey measured general health utility [32]. The Diabetes Treatment Satisfaction Questionnaire (DTSQ) measured treatment satisfaction [33]. The *ADDITION-Plus* lifestyle behaviour change questionnaire included selected cognitions about becoming more physically active [34,35]. The questionnaire included two items to assess perceived behavioural control and intention to become more physically active [36].

### Statistical analyses

Participant characteristics were summarised using means (SD) and percentages. Differences between individuals with and without both self-reported and objective PA data were assessed using t-tests. Univariable linear regression was used to examine the association between PA disparity and socio-demographic, clinical, health behaviour, QoL and psychological characteristics. Beginning with the variable most strongly associated with PA disparity in univariable models, a multivariable model was built via stepwise forward regression. Likelihood ratio tests (LRT) compared models with and without each potential predictor variable and this process was repeated until no new variable improved model fit. Sex, age and trial arm were considered *a priori* confounders and included in all models. A binary ‘PA overestimation’ variable was generated (PA overestimators = 1, PA aware + PA underestimators = 0) and logistic regression was used to examine the association between overestimators and individual characteristics. Correlation coefficients between socio-demographic, health behaviour, clinical, QoL and psychological variables were all *r* ≤ 0.34 and variance inflation factors (VIF) were all ≤4.2. As beta-blocker use and insufficient continuous monitoring of objective PA can affect PAEE estimates, sensitivity analyses were conducted excluding the following individuals: (*i*) 6 participants who wore the Actiheart for less than 48 hours and (*ii*) 103 participants who reported taking beta-blockers. Additionally, as Occupational MET is a key determinant of self-reported PA, [37] sensitivity analyses were carried out which (*iii*) assigned lower MET values to occupational categories; specifically, occupational MET values > 1 were rescaled by 0.75 providing more conservative estimates of activity-specific occupational MET and hence, self-reported Net Activity MET hrs · day^-1^. Finally, to examine if differences in the time frames captured between self-report and objective methods (5 days versus 12 months, respectively) effect validity, (*iv*) one individual from each season (in which objective PA was measured) was randomly selected to form groups of four (n = 100 draws) and between-group correlations were compared with individual-level correlations using random effects regression. Statistical significance was set at a level of *p* < 0.05. All analyses were performed using Stata version 11.2 (Statacorp. College Station, Texas).

## Results

### Descriptive characteristics and physical activity levels of participants

Characteristics of *ADDITION-Plus* participants with complete PA data for analysis (n = 425) are presented in Table 1. Individuals without PA data at one-year were similar to those with complete data for age, sex, socio-economic status and ethnicity (data not shown). The mean (SD) age of participants was 60.3 (7.5) years. The majority of participants were male (63%), Caucasian (97%) and were in managerial/professional or intermediate occupations (41% and 25%, respectively) (Table 1). The majority of participants were overweight (33%) or obese (62%). In just under half of participants (48%, n = 202), mean HbA_1c_ levels were below 6.5% and 78.5% (n = 333) had total cholesterol levels below 5 mmol/l.

**Table 1:** **Descriptive characteristics and physical activity levels at 1 year follow-up in the *ADDITION-Plus *cohort, UK, 2003 to 2007**

**Characteristic**	**Variable**	**Category/scale, units**	**N**	**Mean (SD)**
Socio-demographic	Sex	% Male	425	62.6 (266)^a^
	Age category	42–54.9, years	92	21.6 (92)^a^
		55–59.9	90	21.2 (90)^a^
		60–64.9	81	19.1 (81)^a^
		65–71	162	38.1 (162)^a^
	Ethnicity	% Caucasian	425	97.4 (414)^a^
	Socio-economic category	Managerial/professional	171	40.7 (171)^a^
		Intermediate	104	24.8 (104)^a^
		Routine/manual	145	34.5 (145)^a^
Clinical	BMI	Male, kg/m^2^	264	31.7 (5.1)
		Female, kg/m^2^	159	33.0 (6.0)
	Waist circumference	Male, cm	235	111.9 (13.6)
		Female, cm	148	105.1 (13.1)
Biochemical	HbA_1C_	%	423	6.7 (0.9)
	Total cholesterol	mmol/l	424	4.3 (0.9)
	Systolic blood pressure	mmHg	423	129.9 (17.3)
	Diastolic blood pressure	mmHg	423	76.3 (9.2)
Physical activity	Objective PAEE	kJ · kg^-1^ · day^-1^	425	34.4 (17.0)
		Male, kJ · kg^-1^ · day^-1^	266	37.6 (18.0)
		Female, kJ · kg^-1^ · day^-1^	159	29.0 (13.6)
	Self-reported Net Activity METS_Q_	hours day^-1^	425	12.4 (7.2)
		Male, hours day^-1^	266	13.3 (7.8)
		Female, hours day^-1^	159	11.1 (5.6)
	Self-reported PAEE_Q_	kJ · kg^-1^ · day^-1^	425	22.6 (19.4)
		Male, kJ · kg^-1^ · day^-1^	266	23.7 (21.7)
		Female, kJ · kg^-1^ · day^-1^	159	20.7 (14.6)
Health behaviours	Smoking status at one year	Current	58	13.7 (58)^a^
		Former	209	49.4 (209)^a^
		Never	156	36.9 (156)^a^
	Alcohol consumption	Units · week^-1^	418	7.5 (10.7)
	Total energy	Kcal · day^-1^	424	1738 (493.4)
	Fat	g · day^-1^	424	59.9 (22.4)
	Fruit	g · day^-1^	409	310.8 (218.5)
	Vegetable	g · day^-1^	402	244.9 (138.9)
Quality of life & well being	EuroQol EQ-5D	scale: −0.3 to 1^b^	418	0.8 (0.2)
	ADDQoL	scale: −9 to 9^c^	423	0.9 (1.1)
	SF-36 general health	scale: 1 to 5^d^	425	2.9 (0.9)
	Diabetes treatment satisfaction	scale: 0 to 36^e^	411	30.4 (5.5)
Psychological	Intention	scale: 1 to 5^f^	421	3.5 (0.8)
	Perceived behavioural control	scale: 1 to 5^f^	421	3.6 (0.9)

^a^ % (number).

^b^ Weighted health state index, where dead = 0 and full health = 1.

^c^ Weighted impact of diabetes index, where −9 = maximum negative and +9 = maximum positive impact.

^d^ General health utility scale ranging from, 1 = excellent to 5 = poor.

^e^ Diabetes treatment satisfaction scale, where 0 = very dissatisfied and 36 = very satisfied.

^f^ 5-point Likert-type agree/disagree scale, where 5 represents highest behaviour change beliefs.

### Physical activity energy expenditure

Median (IQR) reported non-sedentary time/reported awake time was 0.28 (0.19 to 0.37). Mean objective and self-reported PAEE levels were 34.4 ± 17.0 kJ · kg^-1^ · day^-1^ and 22.6 ± 19.4 kJ · kg^-1^ · day^-1^, respectively. Mean objective PAEE levels were on average 8.6 kJ · kg^-1^ · day^-1^ lower in females compared to males (95% CI = 5.3 to 11.8 kJ · kg^-1^ · day^-1^, *p* < 0.0001) but there was no difference in self-reported PAEE by sex (difference in means = 3.0, 95% CI = 0.8 to 6.9 kJ · kg^-1^ · day^-1^, *p* = 0.10).

### Physical (PA) activity disparity

Mean ‘PA difference’ (the difference between objective and self-reported PAEE) was on average lower in females than males (Figure 1A & B; difference in means = 5.5, 95% CI = 1.3 to 9.8 kJ · kg^-1^ · day^-1^, *p* < 0.05). Of the 425 participants, 14% (n = 59) fell within the ‘PA Agreement’ zone, 19% (n = 80) were overestimators and 67% (n = 286) were underestimators (Figure 1A & B). The proportion of individuals in the three awareness zones (over- and under-estimator and aware) did not differ significantly by trial arm (χ^2^ = 4.5, *p* = 0.11). Mean objective PAEE levels were significantly higher in PA underestimators compared to PA overestimators or aware (PAEE in kJ · kg^-1^ · day^-1^ for underestimators: 39.9 ± 17.1 1; overestimators: 27.5 ± 15.1; aware: 25.2 ± 11.5; Bonferroni-adjusted pair-wise comparisons, both *p* < 0.0001). ‘PA disparity’ (the absolute difference between objective and self-reported PAEE) captures the magnitude of the difference between the two PA measurements and did not differ between trial arms (difference between mean PA disparity: 1.62 (1.43) kJ · kg^-1^ · day^-1^, t = 1.13, *p* = 0.26). As reported for PA difference, PA disparity was lower in females than males (difference in means = 7.3, 95% CI = 4.5 to 10.2 kJ · kg^-1^ · day^-1^, *p* < 0.0001).

### Individual characteristics associated with PA disparity

In univariable regression analysis male sex and having a routine/manual occupation were positively associated with PA disparity, while older age categories, higher BMI, larger waist circumference and high total daily fruit intake, were inversely associated with PA disparity (Table 2). In multivariable linear regression analysis, men, those in younger age categories, from routine/manual occupations and with lower BMI were more likely to have higher PA disparity (Table 2). Evidence of a linear trend for age and socio-economic status was found, with PA disparity increasing (i.e. a larger difference between objective and self-reported PAEE) from older to younger age classes and from managerial/professional occupations through to routine/manual occupations (LRT of departure from linear trend, *p* > 0.05 in both cases).

**Table 2:** **Association between physical activity disparity and socio-demographic, clinical and health behaviour, quality of life and psychological characteristics in the *ADDITION-Plus *cohort, 2003 to 2007**

		**Univariable**^**a**^			**Multivariable**^**b**^		
**Characteristic**	**Category/scale, units**	**N**	**Regression coefficient (95% CI)**	***p***	**N**	**Regression coefficient (95% CI)**	***p***
**Socio-demographic**							
Sex	♀ = 0, ♂ = 1	425	7.05 (4.24 to 9.87)	0.0001	402	6.45 (3.49 to 9.41)	0.0001
Age category	42–54.9, years	425	1	0.019	402	1	0.012
	55–59.9		−0.77 (−4.94 to 3.40)			0.33 (−3.96 to 4.62)	
	60–64.9		−2.21 (−6.51 to 2.08)			−2.23 (−6.74 to 2.28)	
	65−71		−5.24 (−8.88 to −1.54)			−5.34 (−9.34 to −1.34)	
Ethnicity	Asian/Black = 0, Caucasian = 1	425	0.77 (−3.97 to 5.41)	0.47			
Socio-economic category	Managerial/professional	420	1	0.029	402	1	0.043
	Intermediate		1.92 (−1.66 to 5.51)			1.77 (−1.89 to 5.45)	
	Routine/manual		3.57 (0.33 to 6.81)			4.19 (0.87 to 7.51)	
**Clinical**							
BMI	kg/m^2^	423	−0.31 (−0.56 to −0.061)	0.014	402	−0.39 (−0.65 to −0.13)	0.003
Waist circumference	cm	425	−0.10 (−0.21 to 0.0008)	0.05			
**Health behaviours**							
Current smoking status	Current	58	1	0.63			
	Former	209	2.07 (−2.13 to 6.28)				
	Never	156	1.61 (−2.75 to 5.98)				
Alcohol consumption	Units · week^-1^	418	0.004 (−0.13 to 0.14)	0.95			
Dietary intake	Total energy, Kcal · day^-1^	424	0.001( −0.0018 to 0.0039)	0.46			
	Fat, g · day^-1^	424	0.041 (−0.021 to 0.10)	0.19			
	Fruit, g · day^-1^	409	−0.0074 (−0.014 to −0.001)	0.021			
	Vegetable, g · day^-1^	402	0.0039 (−0.0065 to 0.014)	0.46			
**Quality of life and well being**							
EuroQol EQ-5D	scale, −0.3 to 1^c^	418	1.88 (−4.49 to 8.24)	0.56			
ADDQoL	scale, −9 to 9^d^	423	0.16 (−1.03 to 1.35)	0.79			
SF-36 general health	scale, 1 to 5^e^	425	−0.13 ( −1.67 to 1.41)	0.86			
Diabetes treatment satisfaction	scale, 0 to 36^f^	411	0.071 (−0.18 to 0.32)	0.58			
**Psychological**							
Intention	scale, 1 to 5^g^	421	−0.66 (−2.42 to 1.09)	0.45			
Perceived behavioural control	scale, 1 to 5^g^	421	0.19 (−1.38 to 1.78)	0.80			

Output is taken from linear regression models with PA disparity as the outcome variable.

^a^ Adjusted for the *a priori* confounders sex, age and trial arm.

^b^ Adjusted for sex, age, trial arm, socio-economic category and BMI.

^c^ Weighted health state index, where dead = 0 & full health = 1.

^d^ Weighted impact of diabetes index, where −9 = maximum negative and +9 = maximum positive diabetes impact.

^e^ General health utility scale ranging from 1 = excellent to 5 = poor.

^f^ Diabetes treatment satisfaction scale, where 0 = very dissatisfied and 36 = very satisfied.

^g^ 5-point Likert-type agree/disagree scale, where 5 represents highest behaviour change beliefs.

### Individual characteristics associated with PA overestimation

Logistic regression showed that overestimators were more likely to be in a lower socio-economic category (Table 3). In particular, the odds of being an overestimator were twice as high among those in routine/manual occupations compared with those in managerial/professional occupations (OR = 2.24, 95% CI = 1.22 to 4.12, *p* = 0.009). None of the other variables were significantly associated with PA overestimation (*p* > 0.05).

**Table 3:** **Association between physical activity overestimation and socio-demographic, clinical and health behaviour characteristics in the *ADDITION-Plus *cohort, 2003 to 2007**

**Characteristic**	**Category/scale, units**	**Overestimators % (N)**	**Univariable OR**^**b **^**(95% CI)**	***p***	**Multivariable OR**^**c **^**(95% CI)**	***p***
**Socio-demographic**						
Sex	♀	20.7 (33)	1	0.50	1	0.70
	♂	17.7 (47)	0.84 ( 0.51 to 1.39)		0.91 (0.54 to 1.52)	
Age category	42–54.9, years	14.1 (13)	1	0.55	1	0.51
	55–59.9	18.9 (17)	1.41 (0.64 to 3.12)		1.53 (0.69 to 3.43)	
	60–64.9	22.2 (18)	1.72 (0.78 to 3.82)		1. 77 (0.79 to 3.98)	
	65–71	19.7 (32)	1.52 (0.75 to 3.08)		1.37 (0.66 to 2.81)	
Ethnicity	Asian/Black	18.2 (2)	1	0.97		
	Caucasian	18.8 (78)	1.03 (0.21 to 4.97)			
Socio-economic category	Managerial/professional	12.9 (22)	1	0.009	1	
	Intermediate	21.1 (22)	1.68 (0.86 to 3.31		1.69 (0.86 to 3.31)	0.009
	Routine/manual	24.8 (36)	2.24 (1.21 to 4.12)		2.24 (1.22 to 4.12)	
**Clinical**						
BMI	kg/m^2^	32.6 (4.7)^a^	1.023 (0.98 to 1.07)	0.33		
Waist Circumference	cm	109.7 (11.0)^a^	1.01 (0.99 to 1.02)	0.51		
**Health behaviours**						
Smoking status	Current	22.4 (13)	1	0.35		
	Former	15.8 (33)	0.66 (0.32 to 1.38)			
	Never	21.1 (33)	0.95 (0.45 to 1.99)			
Alcohol consumption	Units · week^-1^	7.6 (9.4)^a^	1.00 (0.98 to 1.03)	0.89		
Dietary intake	Total energy, Kcal · day^-1^	1727 (484)^a^	1.00 (0.99 to 1.01)	0.29		
	Fat, g · day^-1^	59 (22)^a^	0.99 (0.98 to 1.01)	0.76		
	Fruit, g · day^-1^	341 (224)^a^	1.00 (0.99 to 1.01)	0.23		
	Vegetables, g · day^-1^	255 (139)^a^	1.00 (0.99 to 1.00)	0.54		
**Quality of life and well being**						
EuroQol EQ-5D	scale, –0.3 to 1^d^	0.85 (0.2)^a^	1.55 (0.45 to 5.32)	0.48		
ADDQoL	scale, –9 to 9^e^	0.70 (1.0)^a^	0.87 (0.68 to 1.12)	0.27		
SF-36 general health	scale, 1 to 5^f^	2.9 (0.9)^a^	1.10 (0.83 to 1.46)	0.50		
Diabetes treatment satisfaction	scale, 0 to 36^g^	30.4 (5.5)^a^	1.01 (0.97 to 1.07)	0.51		
**Psychological**						
Intention	scale, 1 to 5^h^	3.6 (0.9)^a^	1.31 (0.95 to 1.82)	0.10		
Perceived behavioural control	scale, 1 to 5^h^	3.6 (0.8)^a^	1.09 (0.81 to 1.46)	0.58		

^a^ Mean characteristic of overestimators (SD).

^b^ Adjusted for age, sex and trial arm.

^c^ Adjusted for age, sex, trial arm and socio-economic category (occupation).

^d^ Weighted health state index, where dead = 0 and full health = 1.

^e^ Weighted impact of diabetes index, where −9 = maximum negative and +9 = maximum positive diabetes impact.

^f^ General health utility scale ranging from 1 = excellent to 5 = poor.

^g^ Diabetes treatment satisfaction scale, where 0 = very dissatisfied and 36 = very satisfied.

^h^ 5-point Likert-type agree/disagree scale, where 5 represents highest behaviour change beliefs.

Excluding individuals with less than 48 hours of Actiheart wear (n = 6), or who reported taking beta-blockers (n = 103) did not change these results (data not shown). After assigning more conservative metabolic costs to occupational activity and re-running our series of regression models (linear and logistic), no qualitative difference in the magnitude or the direction of the regression coefficients or odds ratios were found (data not shown). Self-reported and objectively measured PAEE were significantly positively correlated, even when season of objective measurement was adjusted for (Spearman’s correlation coefficients (rho) in men: rho = 0.28, *p* <0.001 and women: rho = 0.27, *p =* 0.006 adjusted for season). Furthermore, very similar correlations were observed when participants were randomly grouped in season-balanced groups of four; the between-cluster rho for self-reported and objective PAEE was 0.24 and 0.23 in men and women, respectively.

## Discussion

To our knowledge, this is the first study to identify characteristics associated with the disparity between objective and self-reported PA on a continuous scale. In this cohort of recently diagnosed T2DM patients from Eastern England, individuals with greater PA disparity were more likely to be male, be in younger age- and lower socio-economic categories and have a lower BMI. Furthermore, when examining the characteristics of those more likely to overestimate their PA (by the PAEE equivalent of 30 minutes of moderate activity per day), overestimators (19%, n = 80) were more likely to be in lower socio-economic categories. Objective PA levels were relatively low in these recently diagnosed T2DM patients. Indeed, compared with a representative sample of 10,000 healthy adults from ten European countries which used the same objective method of assessing PA — *Interact Study*[38] — mean objective PAEE levels were 7.9 kJ · kg^-1^ · day^-1^ lower in our population of recently diagnosed T2DM patients (34.4 and 42.3 kJ · kg^-1^ · day, respectively). Differences in population characteristics may explain some of the between-study PAEE differences; for example, here the average age was 60.3 ± 7.5 years, compared to 53.8 ± 9.4 years in *Interact*. Nevertheless, given the benefits of PA for people with diabetes, these data highlight health promotion opportunities in terms of increasing PA levels.

The association between greater PA disparity and low BMI could be partly explained by the observation that those who underestimate their PA level have substantially higher objective PA levels compared to those who are PA overestimators or PA aware. In addition, leaner individuals may spend less time reflecting on their PA levels than more overweight individuals. Individuals with greater PA disparity were more likely to be male, potentially driven by higher male objective PA levels, coupled with gender differences in the ability to self-assess activity levels in the face of gender norms regarding PA. For example, social desirability/approval may influence female self-reported PA more than males. Several factors may contribute to the observation that PA disparity is greater in younger participants. Social approval is associated with PA underestimation [39] and this factor may be more important in younger age groups. Indeed, post-hoc analyses show that underestimation is most prevalent in the youngest age group (data not shown). In addition, our self-report PA measure may not have adequately captured the activity of this age group, also leading to PA underestimation. Occupational PA is a known key determinant of total PA [37]. Thus, inadequate perception of occupational PA in people with routine/manual relative to managerial/professional occupations may help explain the association between socio-economic category and increased PA disparity. An alternative explanation could be that individuals in lower socio-economic categories may experience more difficulty in completing the questionnaire, resulting in higher PA disparity.

Previous studies assessing ‘PA awareness’ – the difference between an individual’s belief and measured attainment of PA guidelines – have predominantly relied on self-reported and self-rated assessments. We have extended previous work by incorporating an objective measure of PA, which likely reflects true PA more accurately than self-reported or self-rated PA. The different approaches used to classify PA awareness make it difficult to directly compare proportions and characteristics of PA overestimators and underestimators. Previous studies report a lower proportion of underestimators (ranged from 6.1 to 22.5%) and a slightly higher proportion of overestimators (ranged from 15% and 35%) compared with our study (67% and 19%, respectively), with the remaining individuals falling into two other awareness categories [11-14]. In this study, the proportions of individuals in the three awareness groups (over- and under-estimator and aware) did not differ by trial arm suggesting that the intervention was not responsible for the large percentage of under estimators. Reasons for the lower proportion of underestimators in prior studies include the fact that the self-reported PA questionnaire captures specific activities over four domains. Thus, inevitably there will be some activities people engage in that are not included in questionnaires and we expect lower total activity levels from these questionnaires, compared to complete 24-hour recall obtained from continuous wear of objective monitors which capture all activity. Furthermore, the method used here to convert self-reported PA into PAEE removes resting metabolic rate (RMR) to produce estimates that better reflect PA in its own right and not total energy expenditure [40]. Studies which include RMR may produce inflated PA estimates. Despite methodological differences, in terms of the characteristics of overestimators, one previous study found individuals with a family history of T2DM are more likely to be overestimators if they are in lower socio-economic categories [14]. An association between PA overestimation and lower BMI has been reported in healthy adults [11-13] and those at high risk of developing T2DM [14], but similar associations were not shown using PA disparity as an outcome. Further work combining the various awareness measures in one study would be useful.

### Strengths and limitations

The strengths of this study include the use of an objective measure of PA and our derivation of a novel continuous measure of PA disparity based on converting objective and self-report measures to the same units. Our method of objectively assessing PA has been validated against indirect calorimetry during simulated daily activities [41-43] and during free-living against doubly labelled water [44] and likely reflects true PA more accurately than self-report. Use of an objective PA measure to classify PA disparity avoids issues with correlated error arising from use of two self-report measures. Discounting the basal metabolic rate from our self-reported PA data ensures self-reported PA estimates more accurately reflect objective PA. In England, GP registers typically cover ~99% of residents [45] and, as nearly half of GPs approached participated in *ADDITION-Plus,* participants were drawn from a large population-based sample ensuring generalisability to similar locations. Follow-up at one year was also high (93% of living patients).

One limitation of this study is the cross-sectional design which precludes the establishment of causality. Generalisability to ethnically diverse UK populations and/or more deprived areas may be limited due to the recruitment of GPs from a single geographical area, East Anglia England. Observed discrepancies between objective and self-reported PA could partly reflect differences in reference period (4 days for objective and one year for self-reported PA). However, results from sensitivity analyses do not support a major role of between-instrument differences in the time frames captured. Self-reported PA may be underestimated (e.g. if individuals engage in activities that are not listed on the questionnaire) or overestimated (e.g. if the energy costs of activities are overestimated). Indeed, it is possible that assigning MET values to contemporary occupational activities using historical literature overestimated occupational PA in those with routine/manual occupations, which could bias PA disparity with respect to social class (occupationally defined). Sensitivity analyses assigning lower occupational MET values to occupational classes did not alter our findings, suggesting this source of bias is likely to be minimal but cannot be ruled out. Similarly, the scaling of self-reported activity estimates to daily quantities of activity energy expenditure has its limitations, which may be exacerbated by significant proportions of time not accounted for.

What implications do these results have for PA behaviour change interventions? Firstly, given the relatively low level of PA in this sample of individuals with recently diagnosed T2DM, interventions aimed at increasing PA levels are needed and may help improve overall health as well as slow or prevent T2DM progression, as observed in individuals with impaired glucose tolerance [46-48]. Secondly, as high PA disparity (and poor PA awareness) can reduce the effectiveness of interventions aimed at changing PA intentions and behaviour [49], decreasing PA disparity may play an important role in promoting PA. The proportion of people overestimating their PA observed in this study suggests that improving people’s awareness of PA levels may form an important step in interventions aimed at increasing PA. Decreasing PA disparity can be achieved through self-monitoring of, and feedback on PA levels [50,51]. Thirdly, focusing on helping individuals from lower socio-economic categories to become more aware of their PA may be one way of maximising the efficacy of PA interventions.

## Conclusions

The low PA levels amongst T2DM patients early in disease progression suggest a need for interventions to increase PA in this group with high, but modifiable risk. The degree of overestimation suggests that decreasing PA disparity and increasing PA awareness might be an important first step in effective PA interventions [11]. PA interventions should be developed for lower socio-economic groups, which comprise a hard-to-reach population in terms of healthcare equity. Strategies for enabling realistic assessment of PA levels through self-monitoring or feedback warrant further investigation and may help refine and target effective PA intervention programmes.

## Competing interests

All authors declare that they have no competing interests.

## Authors’ contributions

GHL conceived the study question, drafted the analysis plan, conducted the analysis, interpreted the data, drafted and critically revised the manuscript. SG conceived the study question, KS and SG contributed to the analysis plan, interpreted the data and drafted and critically revised the manuscript. SB advised on the statistical analysis, contributed to data interpretation and helped critically revise the manuscript. NW, EvS and SS contributed to data interpretation and helped critically revise the manuscript. All authors read and approved the final manuscript and approved the final version of the manuscript.

## Pre-publication history

The pre-publication history for this paper can be accessed here:

http://www.biomedcentral.com/1471-2458/13/678/prepub

## References

[B1] Nathan DM Buse JB Davidson MB Ferrannini E Holman RR Sherwin R Zinman B Medical management of hyperglycaemia in type 2 diabetes mellitus: a consensus algorithm for the initiation and adjustment of therapy: a consensus statement from the American Diabetes Association and the European Association for the Study of Diabetes Diabetologia 2009 52 1 17 30 18941734 10.1007/s00125-008-1157-y

[B2] Department of Health Start Active, Stay Active: A report on physical activity from the four home countries’ Chief Medical Officers 2011 London: DoH http://www.dh.gov.uk/en/Publicationsandstatistics/Publications/PublicationsPolicyAndGuidance/DH_128209

[B3] Thomas DE Elliott EJ Naughton GA Exercise for type 2 diabetes mellitus Cochrane Database Syst Rev 2006 3 10 15 CD002968 10.1002/14651858.CD002968.pub2 PMC8989410 16855995

[B4] Snowling NJ Hopkins WG Effects of different modes of exercise training on glucose control and risk factors for complications in type 2 diabetic patients: a meta-analysis Diabetes Care 2006 29 11 2518 2527 17065697 10.2337/dc06-1317

[B5] Boule NG Haddad E Kenny GP Wells GA Sigal RJ Effects of exercise on glycemic control and body mass in type 2 diabetes mellitus: a meta-analysis of controlled clinical trials JAMA 2001 286 10 1218 1227 11559268 10.1001/jama.286.10.1218

[B6] The Look AHEAD Research Group Long-term effects of a lifestyle intervention on weight and cardiovascular risk factors in individuals with type 2 diabetes mellitus: four-year results of the Look AHEAD trial Arch Intern Med 2010 170 17 1566 1575 20876408 10.1001/archinternmed.2010.334 PMC3084497

[B7] Andrews RC Cooper AR Montgomery AA Norcross AJ Peters TJ Sharp DJ Jackson N Fitzsimons K Bright J Coulman K Diet or diet plus physical activity versus usual care in patients with newly diagnosed type 2 diabetes: the Early ACTID randomised controlled trial Lancet 2011 378 9786 129 139 21705068 10.1016/S0140-6736(11)60442-X

[B8] Echouffo-Tcheugui JB Sargeant LA Prevost AT Williams KM Barling RS Butler R Fanshawe T Kinmonth AL Wareham NJ Griffin SJ How much might cardiovascular disease risk be reduced by intensive therapy in people with screen-detected diabetes? Diabet Med 2008 25 12 1433 1439 19046242 10.1111/j.1464-5491.2008.02600.x

[B9] Morrato EH Hill JO Wyatt HR Ghushchyan V Sullivan PW Physical activity in U.S. adults with diabetes and at risk for developing diabetes, 2003 Diabetes Care 2007 30 2 203 209 17259482 10.2337/dc06-1128

[B10] Al-Kaabi J Al-Maskari F Saadi H Afandi B Parkar H Nagelkerke N Physical activity and reported barriers to activity among type 2 diabetic patients in the United Arab Emirates Rev Diabet Stud 2009 6 4 271 278 20043039 10.1900/RDS.2009.6.271 PMC2836198

[B11] Lechner L Bolman C Van Dijke M Factors related to misperception of physical activity in The Netherlands and implications for health promotion programmes Health Promot Int 2006 21 2 104 112 16641132 10.1093/heapro/dal011

[B12] Ronda G Van Assema P Brug J Stages of change, psychological factors and awareness of physical activity levels in the Netherlands Health Promot Int 2001 16 4 305 314 11733449 10.1093/heapro/16.4.305

[B13] van Sluijs E Griffin S van Poppel M A cross-sectional study of awareness of physical activity: associations with personal, behavioral and psychosocial factors Int J Behav Nutr Phys Act 2007 4 53 17996060 10.1186/1479-5868-4-53 PMC2186356

[B14] Watkinson C van Sluijs EM Sutton S Hardeman W Corder K Griffin SJ Overestimation of physical activity level is associated with lower BMI: a cross-sectional analysis Int J Behav Nutr Phys Act 2010 7 68 20854659 10.1186/1479-5868-7-68 PMC2954949

[B15] Kipnis V Freedman LS Brown CC Hartman AM Schatzkin A Wacholder S Effect of measurement error on energy-adjustment models in nutritional epidemiology Am J Epidemiol 1997 146 10 842 855 9384205 10.1093/oxfordjournals.aje.a009202

[B16] Day N Wong M Bingham S Khaw K Luben R Michels K Welch A Wareham N Correlated measurement error—implications for nutritional epidemiology Int J Epidemiol 2004 33 6 1373 1381 15333617 10.1093/ije/dyh138

[B17] Griffin SJ Simmons RK Williams KM Prevost AT Hardeman W Grant J Whittle F Boase S Hobbis I Brage S Protocol for the ADDITION-Plus study: a randomised controlled trial of an individually-tailored behaviour change intervention among people with recently diagnosed type 2 diabetes under intensive UK general practice care BMC Publ Health 2011 11 211 10.1186/1471-2458-11-211 PMC3076276 21463520

[B18] Brage S Brage N Franks P Ekelund U Wareham N Reliability and validity of the combined heart rate and movement sensor Actiheart Eur J Clin Nutr 2005 59 4 561 570 15714212 10.1038/sj.ejcn.1602118

[B19] Brage S Ekelund U Brage N Hennings M Froberg K Franks P Wareham N Hierarchy of individual calibration levels for heart rate and accelerometry to measure physical activity J Appl Physiol 2007 103 2 682 692 17463305 10.1152/japplphysiol.00092.2006

[B20] Stegle O Fallert S MacKay D Brage S Gaussian process robust regression for noisy heart rate data IEEE Trans Biomed Eng 2008 55 9 2143 2151 18713683 10.1109/TBME.2008.923118

[B21] Brage S Brage N Franks PW Ekelund U Wong MY Andersen LB Froberg K Wareham NJ Branched equation modeling of simultaneous accelerometry and heart rate monitoring improves estimate of directly measured physical activity energy expenditure J Appl Physiol 2004 96 1 343 351 12972441 10.1152/japplphysiol.00703.2003

[B22] Wareham NJ Jakes RW Rennie KL Mitchell J Hennings S Day NE Validity and repeatability of the EPIC-Norfolk physical activity questionnaire Int J Epidemiol 2002 31 1 168 174 11914316 10.1093/ije/31.1.168

[B23] Ainsworth B Haskell W Whitt M Irwin M Swartz A Strath S O’Brien W Bassett D Schmitz K Emplaincourt P Compendium of physical activities: an update of activity codes and MET intensities Med Sci Sports Exerc 2000 32 S498 S504 10993420 10.1097/00005768-200009001-00009

[B24] James WP Scholfield EC Human Energy Requirements 1990 Oxford: Oxford Medical Publications

[B25] Corder K van Sluijs EM Wright A Whincup P Wareham NJ Ekelund U Is it possible to assess free-living physical activity and energy expenditure in young people by self-report? Am J Clin Nutr 2009 89 3 862 870 19144732 10.3945/ajcn.2008.26739

[B26] Mahabir S Baer DJ Giffen C Clevidence BA Campbell WS Taylor PR Hartman TJ Comparison of energy expenditure estimates from 4 physical activity questionnaires with doubly labeled water estimates in postmenopausal women Am J Clin Nutr 2006 84 1 230 236 16825700 10.1093/ajcn/84.1.230

[B27] World Health Organisation Obesity and overweight. 2011, Fact sheet N°311 2011 Geneva: WHO http://www.who.int/mediacentre/factsheets/fs311/en/ (accessed 14 Aug 2012)

[B28] Bingham S Gill C Welch A Cassidy A Runswick S Oakes S Lubin R Thurnham D Key T Roe L Validation of dietary assessment methods in the UK arm of EPIC using weighed records, and 24-hour urinary nitrogen and potassium and serum vitamin C and carotenoids as biomarkers Int J Epidemiol 1997 26 1 S137 S151 9126542 10.1093/ije/26.suppl_1.s137

[B29] Rabin R de Charro F EQ-5D: a measure of health status from the EuroQol Group Ann Med 2001 33 5 337 343 11491192 10.3109/07853890109002087

[B30] EuroQol EuroQol--a new facility for the measurement of health-related quality of life Health Policy 1990 16 3 199 208 10109801 10.1016/0168-8510(90)90421-9

[B31] Bradley C Todd C Gorton T Symonds E Martin A Plowright R The development of an individualized questionnaire measure of perceived impact of diabetes on quality of life: the ADDQoL Qual Life Res 1999 8 1–2 79 91 10457741 10.1023/a:1026485130100

[B32] Ware JS Kosinski M Gandek B SF-36 Health Survey. Manual and Interpretation Guide 1993 USA: Quality Metric Inc.

[B33] Bradley C The Diabetes Treatment Satisfaction Questionnaire: DTSQ Handbook of psychology and diabetes: a guide to psychological measurement in diabetes research and practice 1994 USA: Harwood Academic Publishers

[B34] Ajzen I The theory of planned behavior Organ Behav Hum Decis Process 1991 50 179 211

[B35] Hardeman W Sutton S Griffin S Johnston M White A Wareham N Kinmonth A A causal modelling approach to the development of theory-based behaviour change programmes for trial evaluation Health Educ Res 2005 20 6 676 687 15781446 10.1093/her/cyh022

[B36] Sutton S French DP Hennings SJ Mitchell J Wareham NJ Griffin S Hardeman W Kinmonth AL Eliciting salient beliefs in research on the theory of planned behaviour: the effect of question wording Curr Psychol 2003 22 3 234 251

[B37] Csizmadi I Lo Siou G Friedenreich C Owen N Robson P Hours spent and energy expended in physical activity domains: results from The Tomorrow Project cohort in Alberta, Canada Int J Behav Nutr Phys Act 2011 8 1 110 21985559 10.1186/1479-5868-8-110 PMC3215175

[B38] The Interact Consortium Validity of a short questionnaire to assess physical activity in 10 European countries Eur J Epidemiol 2012 27 1 15 25 22089423 10.1007/s10654-011-9625-y PMC3292724

[B39] Adams SA Matthews CE Ebbeling CB Moore CG Cunningham JE Fulton J Hebert JR The effect of social desirability and social approval on self-reports of physical activity Am J Epidemiol 2005 161 4 389 398 15692083 10.1093/aje/kwi054 PMC2958515

[B40] Jequier E Thermogenic responses induced by nutrients in man: their importance in energy balance regulation Experientia Suppl 1983 44 26 44 6357848 10.1007/978-3-0348-6540-1_3

[B41] Strath S Brage S Ekelund U Integration of physiological and accelerometer data to improve physical activity assessment Med Sci Sports Exerc 2005 37 11 Suppl S563 S571 16294119 10.1249/01.mss.0000185650.68232.3f

[B42] Thompson D Batterham AM Bock S Robson C Stokes K Assessment of low-to-moderate intensity physical activity thermo genesis in young adults using synchronized heart rate and accelerometry with branched-equation modeling J Nutr 2006 136 4 1037 1042 16549471 10.1093/jn/136.4.1037

[B43] Crouter SE Churilla JR Bassett DR Jr Accuracy of the Actiheart for the assessment of energy expenditure in adults Eur J Clin Nutr 2007 62 6 704 711 17440515 10.1038/sj.ejcn.1602766

[B44] Assah FK Ekelund U Brage S Wright A Mbanya JC Wareham NJ Accuracy and validity of a combined heart rate and motion sensor for the measurement of free-living physical activity energy expenditure in adults in Cameroon Int J Epidemiol 2011 40 1 112 120 20529884 10.1093/ije/dyq098

[B45] Department of Health National surveys of NHS patients General Practice 2002 2002 England: DoH http://webarchive.nationalarchives.gov.uk/20130107105354/http://www.dh.gov.uk/en/Publicationsandstatistics/Publications/PublicationsStatistics/DH_4119522

[B46] Pan XR Li GW Hu YH Wang JX Yang WY An ZX Hu ZX Lin J Xiao JZ Cao HB Effects of diet and exercise in preventing NIDDM in people with impaired glucose tolerance. The Da Qing IGT and Diabetes Study Diabetes Care 1997 20 4 537 544 9096977 10.2337/diacare.20.4.537

[B47] Tuomilehto J Lindstrom J Eriksson J Valle T Hamalainen H Ilanne-Parikka P Keinanen-Kiukaanniemi S Laakso M Louheranta A Rastas M Prevention of type 2 diabetes mellitus by changes in lifestyle among subjects with impaired glucose tolerance N Engl J Med 2001 344 18 1343 1350 11333990 10.1056/NEJM200105033441801

[B48] Knowler W Barrett-Connor E Fowler S Hamman R Lachin J Walker E Nathan D Reduction in the incidence of type 2 diabetes with lifestyle intervention or metformin N Engl J Med 2002 346 6 393 403 11832527 10.1056/NEJMoa012512 PMC1370926

[B49] Godin G Valois P Shephard RJ Desharnais R Prediction of leisure-time exercise behavior: a path analysis (LISREL V) model J Behav Med 1987 10 2 145 158 3612775 10.1007/BF00846423

[B50] Bravata DM Smith-Spangler C Sundaram V Gienger AL Lin N Lewis R Stave CD Olkin I Sirard JR Using pedometers to increase physical activity and improve health: a systematic review JAMA 2007 298 19 2296 2304 18029834 10.1001/jama.298.19.2296

[B51] Michie S Abraham C Whittington C McAteer J Gupta S Effective techniques in healthy eating and physical activity interventions: a meta-regression Health Psychol 2009 28 6 690 701 19916637 10.1037/a0016136

